# Follicular Thyroid Carcinoma Presenting With Endobronchial Metastases: Case Report and Review of Literature

**DOI:** 10.1177/2324709614543691

**Published:** 2014-07-16

**Authors:** Nduche Chika Onyeaso, Aidar R. Gosmanov

**Affiliations:** 1 University of Tennessee Health Science Center, Memphis, TN, USA

**Keywords:** follicular thyroid cancer, endobronchial metastases

## Abstract

Endobronchial metastasis is a rare manifestation of differentiated thyroid cancer. A 79-year-old male was admitted to the hospital with shortness of breath, chest pain, anemia, and weight loss. Computed tomography of chest revealed multiple lung nodules. Bronchoscopy showed an endobronchial lesion in the right upper lobe. The biopsy of the lesion demonstrated neoplastic cells stained positive for thyroglobulin, thyroid transcription factor-1, and cytokeratin-7, consistent with metastatic follicular thyroid cancer. Physical examination revealed a firm fixed thyroid nodule, which was confirmed by thyroid ultrasound. He subsequently underwent total thyroidectomy and neck exploration. Thyroid gland pathology revealed a nodule with features of high-grade follicular thyroid carcinoma. Metastatic thyroid cancer should be considered in workup of pulmonary nodules. We recommend an examination of thyroid gland in patients who present with pulmonary nodules associated with signs and symptoms of malignancy.

## Introduction

Follicular thyroid cancer (FTC) is a differentiated thyroid carcinoma that may metastasize to lung parenchyma. Endobronchial metastasis from FTC is exceedingly rare and is associated with poor prognosis. We describe here a case of a 79-year-old male who presented to us with pulmonary symptoms and was found to have pulmonary nodules. Subsequently, the patient was found to have endobronchial metastases from FTC. In this report, we discuss evolution of the patient’s diagnosis of thyroid cancer and review available literature describing this rare presentation of FTC.

## Case Presentation

**Figure 1.**
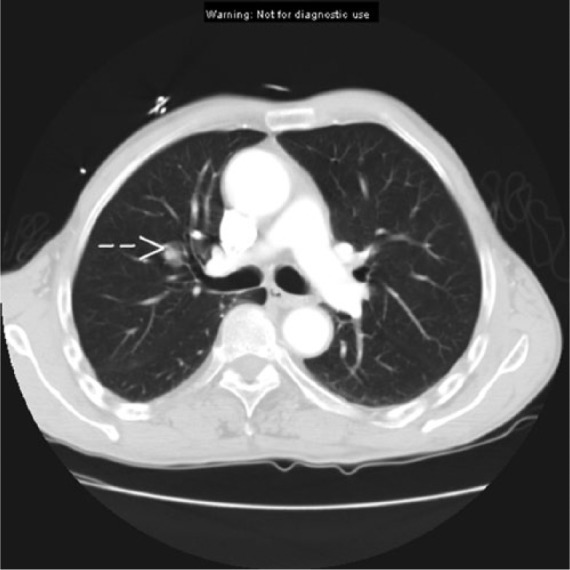
Computed tomography of the lungs demonstrating right pulmonary nodule.

**Figure 2.**
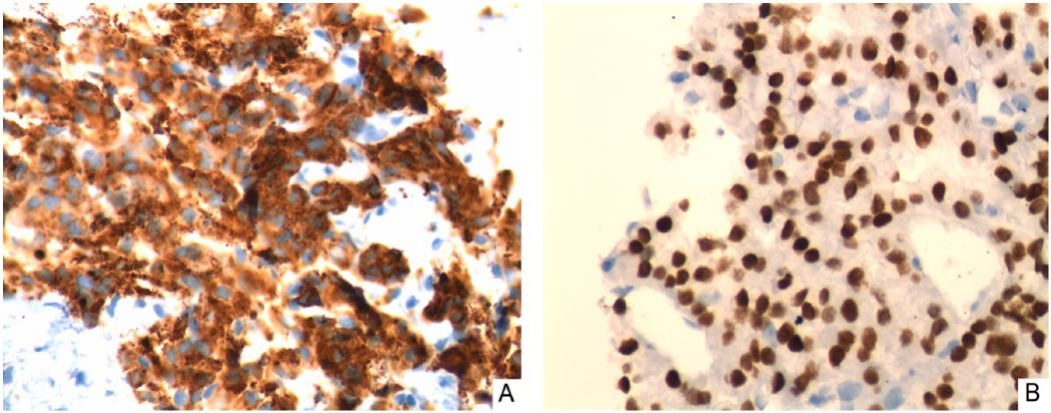
Histological appearance of endobronchial biopsy specimen showing positive immunoperoxidase staining for thyroglobulin (A) and thyroid transcription factor 1 (B) (original magnification 400×).

A 79-year-old African American male presented to a tertiary care hospital with shortness of breath, chest pain, and unintentional weight loss. A computed tomography of chest demonstrated a right lung nodule (Figure 1), and the patient was admitted to the internal medicine service for further workup. His past medical history was significant for a 30-pack year smoking but he quit tobacco smoking 20 years ago. He had no personal or family history of cancer. On initial physical examination, patient was noted to have weight of 55.4 kg with a body mass index of 16.7 kg/m^2^. Vital signs were within normal limits. His thyroid function was normal: thyroid-stimulating hormone (TSH) was 2.08 (0.358-3.74 mIU/mL) and free thyroxine was 1.33 (0.76-1.46 ng/dL). Further inpatient workup that included computed tomography with iodinated contrast and serial plasma troponin levels and electrocardiography studies ruled out presence of pulmonary embolism and acute coronary syndrome. Given past history of smoking and radiological findings of pulmonary nodules, bronchoscopy was ordered. The bronchoscopy demonstrated an endobronchial nodule in the right anterior segment of the right upper lobe and biopsies of the lesion were taken. Pathological evaluation of the lung nodule biopsy revealed neoplastic cells that stained positive for thyroglobulin, thyroid transcription factor-1 (TTF-1), and cytokeratin-7 consistent with metastatic FTC (Figure 2). Staining was negative for cytokeratin-20, prostatic specific antigen, renal cell carcinoma antigen, and synaptophysin. Endocrinology consultation was requested for the further evaluation of FTC.

**Figure 3.**
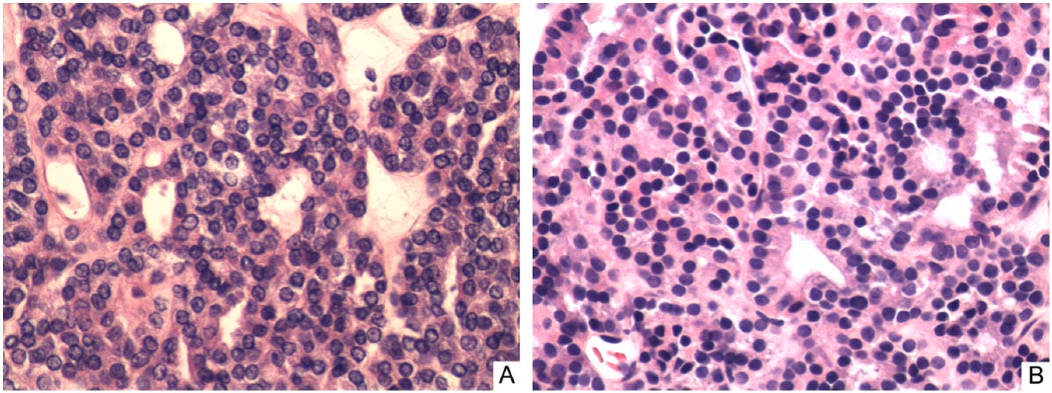
Histological appearance of thyroid tissue (A) and endobronchial biopsy (B) showing similarity between the specimens (hematoxylin–eosin stain, original magnification 400×).

On our examination, the patient had a firm and fixed right thyroid nodule with irregular borders. Additional laboratory investigations demonstrated thyroglobulin level of 657 (2-35 ng/mL) with negative thyroglobulin antibodies. Thyroid ultrasound revealed a 2.3 × 2.2 × 1.8 cm right thyroid solid nodule with regular borders and without microcalcifications or increased vascularity. We subsequently performed an ultrasound-guided fine needle aspiration of the nodule, which revealed presence of colloid and only benign-appearing follicular cells without atypia. At that point, we recommended a thyroidectomy to surgically excise a primary source of FTC that we thought was located in the right thyroid nodule. The patient subsequently underwent total thyroidectomy. Pathological evaluation of thyroid gland revealed widely invasive follicular thyroid neoplasm with capsular and vascular invasion arising from the right thyroid nodule of 3.1 cm in size suggesting the diagnosis of FTC. No lymphatic nodes were dissected during the surgery; therefore, the American Joint Committee on Cancer (AJCC) pathologic stage was T2NXM1 or stage IVC. Areas of the thyroid tumor showed morphology compatible with that seen in the patient’s previous right upper lung nodule biopsy (Figure 3). Postoperative recovery was uneventful. Four weeks after total thyroid hormone withdrawal he achieved TSH level of 60 mIU/mL and thyroid remnant ablation with 152 mCi of I^131^ was performed. Nuclear imaging scans 5 days after the radioactive iodine therapy demonstrated multiple foci of activity in lungs and neck consistent with metastatic thyroid cancer.

## Discussion

The estimated annual incidence of thyroid cancers in the United States is 8.7 per 100 000.^1^ FTC is a differentiated thyroid carcinoma that comprises approximately 10% of thyroid malignancies.^1^ FTC is 3 times more common in females and most frequently found between age of 65 and 69 years. Vascular invasion and metastasis are more common in FTC than papillary thyroid cancer; advanced age, male gender, tumor size, vascular and capsular invasion, and distant metastasis are predictors of poor prognosis in FTC.^2^

Studies demonstrated that mutations of *ras* oncogene could be implicated in the neoplastic transformation of thyrocytes in FTC.^3^ The FTC is metastasized by hematogenous route without involving adjacent lymph nodes that drain the thyroid. Distant metastases occur in approximately 15% of FTCs typically to bones, brain, and lungs.^4^ The lung is the most frequent site of metastatic FTC, and the pathological process usually presents as nodular lesions in lung parenchyma.^2,4-6^ Endobronchial metastases as occurred in our patient are extremely rare with only few case reports in the literature (Table 1).^6-8^ Interestingly, some of patients can present with hemoptysis as initial complaint or can have prior history of FTC (Table 1). The presence of endobronchial metastases usually implies a poor prognosis.^9^ Endobronchial metastases in FTC can develop via several mechanisms. It has been hypothesized that bronchial lesions can occur due to direct invasion of parenchymal lesion, direct extension of mediastinal lesions, transbronchial aspiration, direct lymphatic spread, and/or direct metastasis via the bronchial artery.^10^ The presence of both endobronchial and parenchymal lung lesions may suggest that metastases to parenchymal tissue preceded the development of endobronchial metastatic involvement.

**Table 1.** Summary of Reported Cases of Follicular Thyroid Cancer With Endobronchial Metastases Including the Present Case.

Reference	Age (years) and Gender	Associated Clinical Findings	Bronchoscopy Findings	CT Chest Findings
Ulger et al^6^	77, male	Hemoptysis, right thyroid nodule, right clavicular mass	Tracheal wall polypoid mass	Multiple nodules
Kushwaha et al^7^	58, male	Hemoptysis, recurrent stridor, left thyroid nodule	Tracheal wall polypoid mass	Multiple nodules
Yeo et al^8^	62, female	Cough, total thyroidectomy for FTC 19 years prior to presentation	Endobronchial mass in the right middle bronchus	Multiple nodules
Present case	79, male	Dyspnea, chest pain, right thyroid nodule	Endobronchial mass in the anterior segment of right upper lobe	Multiple nodules

Abbreviations: FTC, follicular thyroid cancer; CT, computed tomography.

Treatment of FTC consists of thyroidectomy and radioactive iodine ablation for tumors that demonstrate radioactive iodine uptake. External beam radiotherapy and chemotherapy are reserved for non-iodine-sensitive refractory cases and/or unresectable cases.^1^ Initial management of FTC in our patient was consistent with the American Thyroid Association guidelines and involved total thyroidectomy with regional lymph node dissection and radioactive iodine thyroid remnant ablation.^1^ With the aggressive management of differentiated thyroid cancer with metastases to lungs, the 5-year survival can reach 70% to 80%.^1^ Therefore, he will require long-term follow-up to monitor for and, if identified, treat persistent disease.

## Conclusion

In summary, this report demonstrates a rare case of FTC that presented with endobronchial metastases. We suggest that patients with signs and symptoms of malignancy and diagnosis of pulmonary nodules of unknown etiology should undergo a more thorough examination of the thyroid gland including its palpation and, if indicated, neck ultrasonography investigation to identify those subjects who may be at risk for metastatic thyroid cancer.
